# Digitalisation of the public sector in Thailand – Insights into Thailand's public sector digitalisation strategy

**DOI:** 10.12688/openreseurope.19868.1

**Published:** 2025-07-31

**Authors:** Anetta Caplanova, Estera Szakadatova

**Affiliations:** 1 Department of Econommics, Bratislava University of Economics and Business, Bratislava Region, Slovakia

**Keywords:** digitalisation, e-Government, Public Sector, public policy

## Abstract

**Background:**

As Southeast Asia embraces digital technology, governments use it to make public services more efficient and accessible. This study explores Thailand’s digitalization efforts, focusing on how government agencies implement e-government services to improve services and reduce costs.

**Methods:**

Using survey data from 288 representatives across central and regional government institutions, the research identifies key focus areas of Thailand’s digital strategy and evaluates its effectiveness through factor and descriptive analysis.

**Results:**

The results show that digital tools are widely used in such sectors as defence, environmental protection, healthcare, and education. The results show that in Thailand, the digital tools contribute to enhancing service delivery, reduce administrative burden, and improve transparency. However, the results show that the financial benefits are frequently underassessed during project planning and evaluation. Only a small number of agencies report to systematically measure the financial returns of ICT projects, despite their long-term potential to strengthen public finances. Challenges to effective digital transformation include uneven digital literacy, low service maturity and limited data integration across agencies. Moreover, concerns about data security and access disparities between urban and rural populations pose further challenges.

**Discussion/Conclusions:**

Thailand’s case demonstrates that digitalization can drive both efficiency and inclusivity, but its success depends on coordinated implementation, robust evaluation and targeted efforts to improve digital skills among citizens and public sector employees.

## Introduction

The digitalization of the Public Sector can lead to considerable financial impacts. By reducing administrative overhead, streamlining public services, and enabling more efficient tax collection, digital tools contribute directly to fiscal sustainability. The financial savings derived from reduced bureaucracy and the automation of routine tasks can be reallocated to critical sectors, such as healthcare, infrastructure, and education.

Digitalization is becoming a crucial way for governments to save money. By automating tasks, reducing paperwork, and reducing bureaucracy, governments can lower their operating costs. For instance, digitizing services such as tax filing and business registration means less need for physical offices and staff, leading to immediate savings. However, the benefits go beyond just cutting costs; digitalization makes public services more efficient and accessible, helps improve transparency, and allows governments to manage resources more effectively. In the long run, going digital can boost a country’s economic growth while also improving the health of its public.

In the evolving area of digitalisation, Southeast Asia emerges, as one of the leaders in the trajectory of technological evolution. As of 2022, the region boasts Internet penetration rates that transcend the global average, with Asia leading at 67.4 percent, more than doubling its 2011 figures ( Statista, 2023a). Southeast Asia’s digital economy has been on a steady growth trajectory in recent years, as the region’s users have continued to top digital engagement in various categories across global rankings, such as time spent online, mobile Internet usage, and mobile app usage ( World Economic Forum, 2023). Since 2016, the number of people online has doubled across six of its largest countries: Indonesia, Malaysia, the Philippines, Singapore, Thailand, and Vietnam ( Statista, 2023b). Consequently, the demand for digital products and services has accelerated rapidly, fueling the growth of the digital economy. This surge in digital connectivity forms the backdrop against which Thailand, a major regional economy, has crafted its approach to Public Sector digitalization.

While the region strides forward, it also faces several challenges. Despite the growing Internet penetration rates, Southeast Asia faces skill disparities that hinder the fulfilment of its digital potential ( OECD, 2023a). These challenges not only lower overall productivity, but also place millions of workers in disadvantaged situations, disproportionately affecting disadvantaged groups.

Over the past decades, digitalization has expanded to an increasing number of sectors, which has also led to the creation and adoption of new business models such as finance, media, tourism, and transportation ( UNDP – Singapore Global Centre, 2023). However, in the context of Southeast Asia, a region marked by its diverse cultures and economic landscapes, the digitalization of the Public Sector represents a pivotal force with far-reaching implications. Digitalization of the Public Sector and economy can also help governments achieve the UN’s Sustainable Development Goals ( United Nations, 2025); however, if digital transformation is not planned and carried out well, it can also contribute to deepening inequalities and related challenges ( UNDP – Singapore Global Centre, 2023). Therefore, it is important that governments and policy makers engage in leading the digital transformation of economies to ensure sustainable development in an environment where the digital transformation potential can be realized, while fostering inclusion and strengthening productivity and economic growth.

This paper aims to comprehensively study the digitalization efforts of the Royal Thai Government. In particular, this study focuses on Thailand's Public Sector Digitalization Strategy, including the fields and policy areas it covers, digital tools, benefits and downsides, and opportunities. Using structural data obtained from surveying public agency representatives, we gained insights into agencies’ experiences with digitalization and e-government services. The survey method was chosen for its effectiveness in providing a snapshot of current attitudes, perceptions, and experiences regarding digitalization efforts.

The results and insights gained from this research can be used to identify gaps and progress in implementing Thailand’s digitalization strategy. In addition, the insights can serve as inspiration for other country governments and policymakers in designing and implementing their digitalization strategies.

## Review of relevant literature

Digitalization is at the forefront of transformative forces that shape societies, economies, and political landscapes globally. Public services often lag behind digitalization; however, they would greatly benefit from it. Digitalization of public services enables instant interaction between citizens and the government, and can contribute to the improvement of inclusivity and accountability ( Frach *et al*., 2017). Increasing the inclusiveness and accountability of public services fosters citizens’ trust, and the use of digital services also shifts the focus of services from the government (i.e., government-centric) to citizens (citizen-centric).

However, to fully benefit from digitalization, governments need to understand what digital in the context of the Public Sector means and how to implement it to improve the quality and efficiency of public services ( Frach *et al*., 2017). Despite the increasing attention on digitalization in the Public Sector, empirical studies have revealed a disparity between intentions and outcomes, considering the efficiency, transparency, and responsiveness of the Public Sector to digitalization ( Maxwell *et al*., 2019).

The Public Sector can be the driver of the transformational and development processes of sectors in an economy; this also applies to digital transformation and digitalization ( Gersonskaya, 2020). Therefore, Public Sector development can have spillover effects, drive the economy, and promote sustainable development. In addition, the Public Sector can contribute to addressing some of the main challenges related to digitalization by developing infrastructure and a regulatory framework to support digital transformation in other sectors. Digital transformation can also have positive effects on the development of leadership and government structures and methods while also improving the quality of public services to citizens and businesses.

In addition, digitalization in general, as well as public and social services, can help countries improve the inclusion of citizens. Ong *et al*. (2023) studied the effect of the digitalization of business processes on financial education in ASEAN countries (i.e., Cambodia, Indonesia, Laos, Myanmar, the Philippines, and Vietnam). The authors use digitalization measures, that is, high-speed broadband, mobile, and cellular subscription, as predictors of financial inclusion. The authors find that digitalization contributes to financial inclusion in the studied ASEAN countries and that digitalization positively affects the accessibility of private businesses to credit provided by their banks.

Digital economies are characterized by the intensive use of ICT for collecting, storing, processing, and transmitting information ( United Nations, 2019). This applies mainly to businesses and companies; however, the government and public sectors also play an important role in this area. Data from industrialized countries have shown that the digital development of economies and businesses operating in them leads, at least in part, to improvements in productivity, which is supported by the supply of goods and services produced by the digital sector via trade ( United Nations, 2019). In addition, a well-developed and resilient ICT sector can lead to the growth of labor productivity.

The globalization and openness of economies can also play an important role in a country’s level of digitalization. Using information globalization as a proxy for information digitalization, Sohag *et al*. (2021) study its effect on institutional quality and agility among ASEAN countries while considering the role of human capital, economic growth, and fiscal spending. They find that public institutions respond positively to information digitalization, highlighting their institutional agility in responding to changing environments. Additionally, human capital, economic growth, and fiscal spending are important determinants of public institutional quality.

OECD (2014) developed a Recommendation on Digital Government Strategies that aims to support countries in their development and implementation. They point out that the use of technology by Public Sector institutions can contribute to the increased effectiveness of public policies and to more open, transparent, innovative, and participatory governments, as well as increase trust in governments. However, the OECD also highlights that some governments do not view technology and digital tools as a means of shaping the outcomes of public governance, even though it has been documented that the current state of technology reinforces existing government processes associated with unsuccessful projects and public dissatisfaction.

Countries ranking highest in digital competitiveness are the USA, Netherlands, Singapore, Denmark, Switzerland, and the Republic of Korea ( World Competitiveness Center, 2024). Australia, Denmark, and the Republic of Korea are some of the most connected countries in the world are Australia, Denmark and the Republic of Korea ( Meyerhoff & Jardanoski, 2020). These countries have high-speed infrastructure, and Internet use is high among both businesses and citizens. In addition, these countries are among the most developed in the use of Information Communication Technology (ICT) in the Public Sector and have achieved high levels of digital transformation of public services. The success of digital transformation in these countries lies in the focus, governance, coordination, and cooperation efforts across government institutions and bodies. Meyerhoff and Jardanoski (2020) found that strong governance with clearly defined roles and responsibilities, cross-sectoral and intergovernmental coordination, and cooperation are important in driving digital transformation. All three countries have demonstrated high levels of inclusiveness across all government levels, society, and user groups (a user-centric approach), which have also contributed to the success of the digitalization agenda and its implementation.

Over the past decades, Danish Governments have aimed to lead e-government development and use and produce efficient personalized social services ( Aagaard & Pedersen, 2022). Successful development can also be attributed to the centralized, neutral, and consensual development of digitalization across the political spectrum. However, strong digitalization also sparked concerns regarding the privacy of citizens and the collection of personal information. Aagaard and Pedersen (2022) argue that in order to protect citizens and to further advance the digitalization agenda, legislation must be adjusted, since algorithmic tools cannot be relied on to solve all tasks.

Even though successful digitalization can be achieved using a consensual top-down approach, the topic will be political. Experience from Denmark shows that digital citizens are governed by discursive, political, and institutional means ( Schou & Hjelholt, 2018). To obtain the right services, the process involves experimenting with different approaches, systems, and methods, which can lead to high costs. The success and legitimacy of digitalization efforts will also depend on citizen trust and social and political accountability ( Aagaard & Pedersen, 2022).

Kuhlmann and Bogumil (2021) highlighted future directions for digital transformation. In a case study of Germany, they found that cooperation and coordination among political and administrative levels are deemed essential in federal systems, acknowledging the importance of variance and diversity in the context of digitalization. This study underscores the legislative role of enhancing user-friendly digital solutions, promoting service bundling, and integrating digital components into administrative processes. Furthermore, it emphasizes the need for an administrative culture that fosters innovation and cross-departmental collaboration, and prioritizes user perspectives.

Furthermore, examining the landscape of local government digitalization, Kuhlmann and Bogumil (2021) identified generational divides in the expectations and usage of online services. While there is a strong demand for online services, satisfaction levels are low, especially among younger citizens and those with lower income. This study highlights the importance of addressing the divide in demographics, expectations, and satisfaction for effective digitalization policies.

Chaipunyathat *et al*. (2019) studied the impact of disruptive technologies on the digital transformation of government services in Thailand, as outlined in the country's master plan for digital economy promotion. This research emphasizes the need for stakeholders, particularly software practitioners and government agencies, to understand the potential effects of digital transformation to enhance the success of software development projects. This study focuses on various emerging technologies, including the IoT, open-source systems, mobile applications, AI, social media, cyber-physical systems, big data, and cloud technology. This research highlights the importance of these technologies in providing equal access to information and digital services, reducing time and opportunity costs.

Addressing the challenges and opportunities in Thailand's digitalization journey, Chaipunyathat *et al*. (2019) conducted a comprehensive analysis of the effects of disruptive technologies on software development. Through interviews, focus groups, and observations, they identified 40 challenges and 35 success factors categorized into laws and regulations, people, software development, enterprise architecture, global influence, and governmental impact. This study emphasizes the potential of disruptive technologies to bridge gaps and provide equal opportunities for information access and digital services.

Digitalization of Thailand is one of the key elements for the country to reach and fulfil its Thailand 4.0 policy. Luangvilai (2019) highlights that the success of digitalization efforts in Thailand, but also in other countries, lies in the selection of appropriate digital technologies, the design of governance, and the implementation of the digital government plan with the aim of improving the well-being of all citizens. Therefore, it is crucial for public officials to have the right digital skills and knowledge, particularly in digital design and enterprise architecture.

### Digital development in Thailand – policy setting

The Thai Government prepared and adopted a two-decade strategy, Thailand 4.0 (stemming from Industry 4.0), aiming for Thailand to become a high-income, developed country by 2036 ( Government of Thailand, 2018; OECD, 2022; The Eastern Economic Corridor Office of Thailand, 2019). The 20-year strategy introduced by the Thai Government in 2018 defines short-term policy objectives aimed at fulfilling the long-term goals of four National Economic and Social Development Plans ( Government of Thailand, 2018; OECD, 2022). To achieve this goal, the strategy focuses on various policy areas, particularly infrastructure and human capital development, so that the country can compete against wealthier, more knowledge-based economies. A great focus of the policies is also on innovation and the fields of automation, robotics, logistics, or digital transformation and development.

Thailand 4.0 represents the fourth development plan adopted by the government and builds on previous development plans that focused on agriculture (Thailand 1.0), light industry (Thailand 2.0), and heavy industry (Thailand 3.0). Within strategy Thailand 4.0, the government aims to build and develop a high-tech economic zone in eastern Thailand, that is, the Eastern Economic Corridor (EEC), consisting of provinces Rayong, Chonburi, and Chachoengsao. Various policy initiatives focus on the development of infrastructure and human resources and attract investment into the EEC.

In terms of digitization, Phase 1 of the Thailand 4.0 strategy will aim to lay foundations for the digital infrastructure, including e-Government services; Phase 2 will aim to foster inclusiveness, ensuring that all citizens and stakeholders can benefit from digitalization; Phase 3 will enable full digital transformation, driving the economy with digital technology and innovation; and Phase 4 will establish Thailand as a global leader in digital technology and innovation ( Ministry of Information and Communication Technology, 2016).

The Thai Government set out an ambitious plan to transform Thailand into a developed country by the year 2036; however, to achieve the set long-term goals, the Thai Government will have to implement and adapt the digitalization agenda on a wider scale and make it more efficient ( OECD, 2022).

### Digital Economy and Society Development Plan

The plan aims to transform Thailand into a country that uses digital technologies in various areas to develop infrastructure, innovation, data, human capital, and other digital technologies and tools to support the growth, stability, and sustainability of the economy ( Ministry of Information and Communication Technology, 2016). One of the plan’s strategies is the transformation into a digital government (strategy number 4), which aims to create an open government that facilitates citizens and businesses.

In addition, led by the Digital Government Development Agency, Thai Governmental organizations in cooperation with Burapha University developed an initiative (Digital Local Support Centre Project for Administration and Public Service) that aims to improve the utilization of digital platforms by local governments and support local administrative organizations in integrating digital technology into their work ( Ministry of Information and Communication Technology, 2016; Saffa, 2024). Digitalizing local government systems can help improve public service delivery and efficiency, reduce travel time, and improve the accessibility of government services for citizens. The aim of the initiative is to place citizens’ needs at the center of digital development. Furthermore, to integrate the public service system, a central platform (i.e., Digital Local System) has been developed to improve the management of electronic archives and documents, which is an important part of the Digital Local System.

The Digital Local System also contributes to the alignment of government practices, improving the efficiency and transparency of regional and local governments. The Digital Local Support Center Project (Source) will also support the development of digital skills among public officers and personnel across different government agencies to contribute to the effective implementation of the project and digitalization efforts of the Public Sector and Government services.

Thailand’s digital transformation plan for 2024 aims to strengthen the country’s digitalization and competitive advantage at the international level ( Saffa, 2024). The strategy focuses on seven main areas: fostering innovation, enhancing public services, and stimulating economic growth through the strategic implementation of digital technologies.

In addition, the strategy will focus on the development of Thailand’s AI infrastructure and a large language model (Thai LLM), which will contribute to the promotion of ethics, governance, and the regulation of AI ( Saffa, 2024). By enabling more efficient and effective communication, data, and information processing, the use of AI in the Public Sector is aimed at benefitting different sectors such as education, healthcare, and customer services.

A country review carried out by the OECD (2022) suggests that Thailand is committed to developing and implementing an open and connected government plan, which the government demonstrated by the introduction of the Digital Government Development Plan for the years 2017–2020. Furthermore, Thailand introduced and implemented legal instruments related to digital governments, tools enabling stakeholder engagement, and public access to information. The coordination of Thailand’s digital government agenda is assigned to various ministries that highlight the government’s commitment to the digitalization agenda at the highest political level.

However, to benefit from the success of the open Government, Thailand must invest in building an open culture, proactively engage with stakeholders across different sectors and spheres, including citizens, and use digitalization as the main tool for open and digital government.

The OECD (2022) also highlights that Thailand lacks several areas of digitalization, such as the planning and monitoring of ICT investments and expenditures, and the adoption of tools and processes that contribute to the transformation to a digital government. Thailand would benefit from improving these areas, as it would contribute to a more coherent adoption of the digitalization agenda across different policy areas, levels of government, and projects.

## Data and methods

### Data

This section presents the data collected for the analysis of the digital government services of The Royal Thai Government from the perspective of government agency representatives from different fields and industries. In addition, the data provided us with a qualitative overview of the effectiveness of digital government tools and services.

The data were collected using a cross-sectional survey and were predominantly qualitative in nature, allowing us to gain a deeper understanding of the types of e-government and digital services used at different government levels in Thailand. The survey was based on the OECD Digital Government Survey ( OECD, 2023b). Data were collected through a structured survey questionnaire administered online. The questionnaire was designed to capture various dimensions of Thailand’s e-government initiatives, including perceived effectiveness, effectiveness, challenges, agency experiences, and the overall impact of the digitalization strategy. The data were collected from Government and Public Sector agency officials in different fields, such as agriculture, transport, local administration, and industry, as well as officials from ministries (e.g., Ministry of Digital Economy and Society). The sample size included 288 respondents.

The summary data in Table 1 show that the majority (i.e., almost 86 percent) of the respondents were employees and representatives of central government institutions. None of the respondents were employees of government institutions at the local level. Considering the number of employees, 61 percent of respondents were employees of large institutions with more than 250 employees, while 22 percent of respondents worked for medium-sized institutions and 17 percent worked for small institutions with less than 50 employees.

**Table 1.** Survey sample representation of Government agencies.

		Number	Percentage [%]
*Government level*	*Central*	247	85.76
	*Regional*	41	14.24
	*Local*	0	0
*Number of employees*	*Less than 50 employees*	50	17.36
	*50–249 employees*	63	21.88
	*More than 250 employees*	175	60.76

*Source: Authors’ own computations based on the results of the conducted survey ( Čaplánová & Szakadátová, 2023)*.

### Methods

This section describes the methodology used to analyse the collected data. The methodological approach includes factor and descriptive analyses to assess the focus areas of Thailand’s national digital strategy e-government services.

The collected survey data were obtained with the informed written consent of participants, who were provided with a detailed participation information sheet outlining the purpose of the research, data confidentiality and GDPR compliance. The data were collected during a two-month horizon (between March 1 ^st^ and May 3 ^rd^, 2024) and respondents were able to complete the questionnaire in their own time. The data were analysed using factor analysis, which was complemented with descriptive analysis, which allowed us to gain deeper insights into the state of digitalization of the public sector and its services in Thailand.

Factor analysis was used to identify the underlying dimensions or factors that influenced the perceptions and effectiveness of Thailand's e-government initiatives. Factor analysis allowed us to reduce the large set of observed variables (survey responses) into a smaller number of factors, making it easier to understand the core components driving the digitalization strategy's focus areas across agencies, as well as the methods used to assess the benefits of digitalization projects.

A factor analysis was conducted using principal component analysis to maximize the variance of the loadings of each factor. This method was selected to achieve a clear and interpretable factor structure. The suitability of the data for the factor analysis was assessed using Cronbach’s alpha. Only factors with eigenvalues greater than 1 were retained in the analysis, and items with high loadings on each factor were analyzed to determine the underlying themes.

The analysis was complemented by summary and descriptive statistics, which were used to summarize the data and provide a detailed overview of the survey results. The analysis provides insights into the development of Thailand’s digital government services, as well as their effectiveness. In addition, descriptive analysis allowed us to gain deeper insights into the qualitative data and agency representatives’ perceptions and experiences.

## Results

Survey responses also suggest that the financial benefits of digital transformation are often overlooked in the initial planning stages. However, a thorough cost-benefit analysis reveals that digitizing public services, such as tax filings, healthcare claims, and business registrations, can significantly reduce government expenditure. This can lead to long-term financial savings across sectors and improve the fiscal accountability. For instance, the implementation of e-government services reduces the need for physical infrastructure, leading to budget savings and increased efficiency.

The digitalization of Public Sector services and digital governments can positively contribute to the development of societies as well as their skills development. In Thailand, many citizens believe that the country’s digital transformation will lead to improved public development, welfare, and health ( The Economist, 2022).

The Thai national strategy for the digital government covers different areas, showcasing the government’s commitment to bringing digital services to citizens in different areas and making public services more easily accessible to them. Apart from covering general digital services, the digital government covers policy areas such as defense, public order and safety, economic affairs, environmental protection, housing, healthcare, recreation, culture, religion, education, and social protection. In addition, the digital government also supports intra-governmental services, such as international relations, infrastructure, and agriculture, and also enables Governmental and Public Sector agencies to report results and communicate more efficiently. Respondents from the Department of Agriculture highlighted that digital technology drives R&D in plants, agricultural machinery, and agricultural product certification in a stable and sustainable manner.

Thailand also has a national portal for citizens and businesses that mainly provides links to specific websites of relevant authorities; that is, it centralizes information without duplication of services. The portal also provides access to services uniquely provided by the authority in charge of the portal.

Table 2 summarizes the factor loadings obtained from the factor analysis of the policy areas covered by Thailand’s national digitalization strategy by the surveyed agencies. Based on the Eigen criterion, only four out of nine factors were retained, as their eigenvalues were higher than 0; however, we assume that only the first three factors are meaningful, as the eigenvalue of factor 4 is close to 0 (i.e., 0.06 (see Appendix Table 1).

**Table 2.** Results of the factor analysis of the policy areas covered by Thailand’s national digitalisation strategy.

	Factor 1	Factor 2	Factor 3	Factor 4
*General public services*	0.3221	0.0440	-0.2164	0.0688
*Defence*	0.7248	-0.3332	0.0336	0.0361
*Public order and safety*	0.6811	-0.3184	-0.0320	0.0274
*Economic affairs*	0.4790	0.0915	-0.0538	-0.1604
*Environmental protection*	0.8044	-0.0306	0.0206	-0.0821
*Housing and community amenities and Health*	0.7525	0.2837	0.0353	0.0512
*Recreation, culture religion and Education*	0.7434	0.2010	0.0213	0.1014
*Social protection*	0.6996	0.0854	0.0822	-0.0495
*Other*	-0.1018	-0.0224	0.2320	0.0333

*Source: Authors’ own computations based on the results of the conducted survey ( Čaplánová & Szakadátová, 2023)*.

 The results in Table 2 show that Factor 1 has relatively high positive loadings (i.e., higher values on this factor correspond to greater awareness of these areas by the agencies), particularly for the policy areas defense (0.72), environmental protection (0.80), housing and community amenities and health (0.75), and recreation, culture, religion, and education (0.74). This suggests the coverage of broader social and environmental policy areas by the surveyed public agencies. The survey responses also suggest that the financial benefits of digital transformation are often overlooked during the initial planning stages. However, a thorough cost-benefit analysis reveals that digitizing public services, such as tax filings, healthcare claims, and business registrations, can significantly reduce government expenditure. This can lead to long-term financial savings across sectors and improve the fiscal accountability. For instance, the implementation of e-government services reduces the need for physical infrastructure, leading to budget savings and increased efficiency.

Factor 2 had mixed, positive, and negative loadings with a lower magnitude. Factor 2 shows low negative loadings (i.e., higher values on this factor correspond to less awareness of these areas by the agencies); however, the results show higher factor loadings on policy areas defense (-0.33) and public order and safety (-0.32), as well as housing and positive loading on community amenities and health areas (0.28), suggesting a broader coverage of these areas of Thailand’s national digitalization strategy by surveyed public agencies, as well as on security and public safety-related policy areas.

Factor analysis showed that Factor 3 did not have very high factor loadings. The results show that the area of public services has a slightly higher negative loading, suggesting a focus of surveyed agencies in this area of Thailand’s digitalization strategy.

Cronbach’s alpha was 0.8471, suggesting internal consistency of the coefficients. Cronbach’s alpha above 0.7 highlights the reliability of the factors identified from the data (see Appendix Table 2).

The majority of respondents (66 percent) reported that the Government and the Government agencies monitor progress towards achieving the goals of the digital government strategy. Approximately one-fifth of the agency representatives reported that the full potential direct financial benefits of less than 50 percent of current ICT projects are being measured and followed up centrally. Only 10 agency representatives reported that the performance of 50–75 percent of ICT projects is monitored at the central level, and 19 agency representatives reported that the financial benefits of 75–100 percent of ICT projects are being measured at the central level; these respondents represent mostly agricultural, transport, and trade agencies. 72 percent of the agency representatives reported that they were not aware of the government measuring the financial benefits of ICT projects.

Table 3 reports the results of the factor analysis of the methods of assessing the benefits of realised ICT projects by relevant Thai agencies and authorities. Based on the eigen criterion, only 3 out of 6 factors were retained, as their eigenvalue was higher than 0 (see Appendix Table 3). The results show that factor 1 has high positive loadings in using the output in concerned authorities to assess the direct benefits of realised ICT projects. In addition, high positive loadings can be observed in service quality in concerned authorities to measure the direct benefits of ICT projects.

**Table 3.** Results of the factor analysis of the assessment of benefits of realised ICT projects.

	Factor 1	Factor 2	Factor 3
*Increase in service quality in concerned authorities*	0.7782	-0.3118	-0.1259
*Increase in output in concerned authorities*	0.8112	-0.3117	-0.0831
*Budget reductions in concerned authorities*	0.6720	0.2708	-0.2211
*Staff reductions in concerned agencies*	0.5929	0.4214	-0.0692
*Staff reallocation across the government agencies*	0.6432	0.1472	0.2599
*Realised financial benefits can be used at the discretion of the concerned entities*	0.6363	-0.0487	0.2953

*Source: Authors’ own computations based on the results of the conducted survey ( Čaplánová & Szakadátová, 2023)*.

Factor 2 shows low positive and negative loadings; however, a relatively high positive factor is observed when using staff reductions in concerned agencies as a method to measure the benefits of implementing ICT projects. Factor 3 showed low factor loadings; however, using realized financial benefits as a method for assessing direct financial benefits had slightly higher loadings. Cronbach’s alpha was 0.8411, suggesting internal consistency of the coefficients (Appendix Table 4). The survey responses also suggest that the financial benefits of digital transformation are often overlooked during the initial planning stages. However, a thorough cost-benefit analysis reveals that digitizing public services, such as tax filings, healthcare claims, and business registrations, can significantly reduce government expenditure. This can lead to long-term financial savings across sectors and improve the fiscal accountability. For instance, the implementation of e-government services reduces the need for physical infrastructure, leading to budget savings and increased efficiency.

In addition, government agencies use other methods to measure the direct benefits of ICT projects, such as better data availability and accuracy (e.g., big data database), reduction in burden, time, and resource savings. Agencies also have key performance indicators to ensure that the benefits of ICT projects are achieved, and agencies can also be awarded the Public Sector Management Quality Award (PMQA). There is also one stop shop and services aimed at helping reduce the burden on the public by having to contact multiple agencies. Almost all respondents reported that they were unaware of government agencies measuring the direct benefits of ICT projects for citizens and businesses.

The survey results show that the most cost-effective public service delivery channel is the national service online portal, followed by agency portals or websites, and mobile platforms (such as applications, SMS, MMS, or online services designed for mobile devices). Surveyed agency representatives reported that less cost-effective public service delivery channels are shared service centers, call centers, and services, while the least cost-effective public service delivery channel is traditional delivery methods, such as filling out paper documents.

The main identified barrier for increasing the number of mandatory online services is the low maturity and quality of online service delivery and that not all users have sufficient ICT skills – data show that there is an urban-rural disparity in citizens’ ICT skills ( UNICEF & ASEAN, 2021). Given the lack of ICT skills, respondents reported that citizens were concerned about the security of their personal data. In addition, agency representatives identified the channel of public service delivery methods as citizens’ rights; therefore, they could not be mandated to use online services. It has also been highlighted that Thailand still lacks a comprehensive service system developer because the existing system is not fully functional and has problems in usage, and data integration between agencies is difficult, which hinders the development of services that require shared data between agencies.

The majority of respondents (53 percent) reported that Thailand has a legally recognized digital identification mechanism. Approximately 32 percent of respondents reported that they did not know that the digital identification mechanism was used in Thailand, while 14 percent of respondents reported that Thailand does not have a legally recognized digital identification mechanism in place. Most of the respondents who responded negatively were officials from institutions at the central government level, and their agencies had more than 250 employees. The areas where these respondents worked included industry, agriculture, trade, and road and highway infrastructure. These results suggest that awareness and use of the digital identification mechanism should be improved. 

Thailand has a legally recognized digital identification mechanism, which predominantly uses a digital identity verification and authentication system in the ThaiID application. Moreover, it is also used at the executive level to approve certain operations, such as electronic document systems or leave-approval systems, to sign official documents, or it is used in the electronic document system. Entrepreneurs can also use digital signatures to submit requests for the documents required for the export and/or import of goods.

Regarding the services the digital identification mechanism can be used for, approximately 44 percent of respondents responded that the digital identification mechanism can be used for public services provided at the central government level, while only 10 percent of respondents reported that the digital identification mechanism can be used for public services provided by subnational governments. However, the majority of officials from regional governments indicated that digital identification mechanisms cannot be used for public services provided by subnational governments. About a fifth of the respondents reported that the digital identification mechanism can be used for private sector services (e.g., in banks). In addition, the responses highlighted that digital identification can be used for communication within agencies.

The results show that Thailand has developed e-government services across different Public Sectors, which aligns with Thailand’s digitalization strategy. In addition, to improve the quality of these services and public services, the government also focuses on the development of the digital and technology skills of Public Sector employees. Public sector employees are offered IT and ICT training and skills development programs (including e-learning), but also specific programs to develop their digital skills to drive the digital government and foster their innovative and design thinking. The development programs are also supported by the Digital Government Development Agency, and employees can also gain certification.

Moving to digital systems in the public sector comes with upfront costs, such as investing in new technology, developing software, and training staff to use new tools. These steps are necessary to obtain everything up and run. However, its long-term benefits make it a worthwhile option. Digitalization can reduce operational costs by automating routine tasks, reducing paperwork, and requiring fewer physical offices. It can also help to increase government revenue by making tax collection more efficient. In the long run, these changes can save money and improve the public sector’s financial health.

## Discussion and conclusion

This research provides a comprehensive understanding of the global and regional landscape, that is, of Thailand, of Public Sector digitalization efforts and strategies, offering valuable insights into challenges, factors influencing digitalization, and future directions. The analytical part of this study focuses on the State of Government and Public Sector digitalization in Thailand. Responses from Public Sector agency representatives highlighted the areas covered by the Thai national digitalization strategy, their effectiveness, and opportunities for development.

The results show that the Thai national digitalization strategy covers policy areas defense, environmental protection, housing, community and health care services, recreation, culture, religion, and education. The survey results show that the Government and Government agencies monitor progress towards the achievement of digital strategy goals. However, only a minority of respondents reported that the direct financial benefits of ICT projects are being measured and followed centrally. The benefits of digitalization are measured predominantly by assessing the increase in the output and service quality of concerned authorities as well as budget savings.

In addition, the results show that agency employees and representatives are encouraged to develop their digital literacy and skills to align with the needs of digital progress and transformation. However, for digital transformation to fully achieve its goals and contribute to the technological development of society and the improvement of the quality of public services and welfare, it is also important that the digital skills of citizens are improved. The data show stark disparities in digital skills across socio-demographic groups in the ASEAN region, emphasizing the need for targeted efforts to enhance digital literacy, particularly in rural areas and among ethnic minorities ( OECD, 2023a and UNICEF & ASEAN, 2021). Furthermore, there is an infrastructure divide between urban and rural areas ( OECD, 2023a).

Therefore, without efforts to improve the digital infrastructure and literacy of citizens, only those living in urban areas will benefit from digital transformation to the greatest extent possible. Otherwise, the digital divide will hinder development and deepen inequality between regions, particularly in urban and rural areas. For Thailand and Southeast Asian countries to fully benefit from the economic growth and development driven by the digital economy, stakeholders (i.e., governments, citizens, businesses, NGOs, and investors) must cooperate to foster digital inclusion and participation.

Digitalization has the potential to significantly impact public finance in Thailand. By automating tasks and reducing paperwork, the government can save money on administrative costs and reduce the need for physical offices. Digital tools can also help boost revenue by making tax collections more efficient and reducing fraud. Additionally, access to real-time data on spending will allow for better budget planning and more transparent financial management. If fully implemented, digitalization could not only improve Thailand’s fiscal health, but also build greater public trust in how government funds are used.

The conducted research also has drawbacks, particularly owing to the nature of the survey data, which can negatively affect its reliability. In addition, not all agency representatives may be informed equally or adequately about Thailand’s national digitalization strategy; therefore, the results may be skewed. However, given the comprehensive structure of the survey, we believe that the collected data are sufficient for understanding the Thai Government’s digitalization efforts.

Future research will focus on further deepening the knowledge and understanding of digital transformation in Thailand and other Southeast Asian countries. We will also aim to carry out the survey in other countries so that we can compare and benchmark governments’ digitalization efforts. Future research can also focus on the comparison of progress towards government digitalization among countries in different geographical locations.

## Ethical approval

Ethical approval for the ODDEA project was obtained through the appropriate ethics procedures. The research was reviewed and approved by the ODDEA project ethics expert at Bratislava University of Economics and Business, Prof. Durgesh Tripathi, PhD on April 2, 2025. Specific reference permit number is not available as the ODDEA project does not utilise a reference permit numbering system.

## Consent statement

All participants in this study provided informed consent prior to their inclusion. The consent process was conducted in accordance with relevant guidelines and regulations. The consent was provided in written form by starting the survey. Where applicable, consent included permission for the anonymised data to be published and shared openly, in line with the Open Research Europe platform's policies on open data and transparency.

## Data Availability

*Zenodo:* Digital Government Performance Thailand, https://doi.org/10.5281/zenodo.15626758. ( Čaplánová & Szakadátová, 2025) *This project contains the follwoing underlying data:* Digital government survey ODDEA(1-8).xlsx Digital government survey ODDEA.pdf Digitalisation of the Public Sector in Thailand Appendix.pdf Information sheet ODDEA.pdf *The data underlying this article are available in Zenodo Data Repository and can be accessed under the terms of the Creative Commons Attribution 4.0 International (CC BY 4.0)*.
