# Supplementary material for: Digitalisation of the public sector in Thailand – Insights into Thailand's public sector digitalisation strategy
Source: Open Res Eur. 2025 Dec 2;5:209. Originally published 2025 Jul 31. [Version 2] doi: 10.12688/openreseurope.19868.2 (PMC12739367; doi:10.12688/openreseurope.19868.2)
Supplement: Peer review history

# Reviewer report: Reviewer response for version 2

**DOI:** 10.21956/openreseurope.23800.r65330

**Published:** 2025-12-26

**Authors:** Oana Staiculescu

**Affiliations:** 1 Universitatea din Craiova, Craiova, Romania

The authors have addressed the key concerns raised in the initial review in a thorough and satisfactory manner. In particular, the revised version now clearly moderates earlier claims regarding the financial impacts of digitalisation and correctly frames these effects as perceived benefits rather than empirically verified outcomes. The limitations related to the concentration of respondents at the central and regional levels and the absence of local government representation are now explicitly acknowledged, improving the study’s methodological transparency. Furthermore, the factor analysis has been significantly improved through the clear conceptual labelling of factors and a more nuanced interpretation of the lower explanatory power of Factors 2 and 3. These revisions substantially strengthen the analytical rigor and internal coherence of the manuscript. I consider the revised article suitable for approval.

If applicable, is the statistical analysis and its interpretation appropriate?

Partly

Is the study design appropriate and is the work technically sound?

Partly

Is the work clearly and accurately presented and does it engage with the current literature?

Yes

Are the conclusions drawn adequately supported by the results?

Partly

Are sufficient details of methods and analysis provided to allow replication by others?

Yes

Are all the source data and materials underlying the results available?

Yes

Reviewer Expertise:

Public Administration, Digitalization, E-democracy, Public Management, Leadership, Public Sector Accounting, and Economics.

I confirm that I have read this submission and believe that I have an appropriate level of expertise to confirm that it is of an acceptable scientific standard.

# Reviewer report: Reviewer response for version 2

**DOI:** 10.21956/openreseurope.23800.r65430

**Published:** 2025-12-24

**Authors:** Rakibul Hasan Chowdhury

**Affiliations:** 1 Trine University, Angola, Indiana, USA

**Summary of the Article**

 This manuscript investigates Thailand’s public-sector digitalisation strategy based on survey data from 288 representatives of central and regional government agencies. The study examines:

- The policy areas covered by Thailand’s digitalisation strategy
- The extent of digital adoption across government services
- Methods used by agencies to evaluate ICT project benefits
- Perceived barriers, including digital skills, data integration, and security concerns
- Awareness and utilisation of Thailand’s digital identification system
- Perceived cost-effectiveness of service delivery channels

Factor analysis is used to group policy domains and ICT evaluation practices, and descriptive analysis provides insight into perceptions of digitalisation outcomes. The study positions Thailand’s efforts within the broader ASEAN and global digitalisation context. The Discussion reflects on implications for digital inclusiveness, public finance, and policy alignment.

 The work is timely and relevant, especially given the rapid digitalisation processes underway in Southeast Asia. It offers valuable descriptive insights into government perceptions and highlights structural challenges. Nonetheless, several methodological gaps and issues with factor-analysis reporting limit the robustness of the analytical claims.

**General Assessment**

 The manuscript contributes meaningfully to the understanding of Thailand’s public-sector digitalisation landscape. The topic is relevant, the dataset is openly available, and the authors present a good deal of contextual and descriptive analysis.

 However, several areas require clarification and strengthening before the work can be considered scientifically sound. These include:

- Missing or incomplete statistical reporting
- Methodological inaccuracies (e.g., incorrect eigenvalue criterion)
- Lack of key diagnostics supporting factor analysis
- Occasional conceptual overstretching regarding financial implications
- Need for more coherent structuring of results

With revisions, the article can provide a stronger empirical and analytical foundation for its conclusions.

**Assessment of the Key Criteria**

**1. Clarity of Presentation and Engagement with Current Literature**

**Rating: Partly**

**Strengths**

- The literature review is extensive and well-sourced.
- It discusses global, ASEAN, and Thai-specific scholarship.
- It contextualises digitalisation in terms of governance, inclusion, and institutional quality.

**Issues and Improvements**

1. **Structural clarity**:
  The literature review is lengthy but lacks a clear conceptual structure. Organizing it into themes (e.g., digital government performance, governance & coordination, digital skills & inclusion, institutional impacts) would make it more analytically coherent.
2. **Insufficient linkage to results**:
  The review introduces frameworks (OECD, ASEAN, digital competitiveness indices) that are not explicitly revisited in the Results or Discussion.
3. **Opportunity for stronger theoretical anchoring**:
  The study could be substantially strengthened by integrating:
  - The OECD Digital Government Policy Framework
  - The UN Digital Government Model
  - Sociotechnical or institutional capacity frameworks
  - Technology–Organization–Environment (TOE) theory

These additions would improve conceptual consistency and analytical depth.

**Required for scientific soundness:**

 ✔ Reorganize the literature into thematic sections

 ✔ Add explicit conceptual or theoretical frameworks guiding empirical interpretation

**2. Study Design and Technical Soundness**

**Rating: Partly**

**Strengths**

- Cross-sectional survey design is appropriate for capturing perceptions.
- The research questions align well with the descriptive nature of the data.

**Critical Issues**

1. **Incorrect eigenvalue criterion:**
  The authors state that factors were retained because eigenvalues were **greater than 0**, but the widely accepted **Kaiser criterion requires eigenvalues > 1**.
  This is a methodological error and must be corrected.
2. **Missing diagnostics for factorability:**
  The manuscript does not report:
  - **Kaiser–Meyer–Olkin (KMO) measure**
  - **Bartlett’s test of sphericity**
  These are required to justify conducting factor analysis.
3. **Ambiguous rotation method:**
  It is unclear whether Varimax, Promax, or another rotation was used. Clear reporting is required.
4. **Interpretation of factors with low or mixed loadings:**
  Factors 2 and 3 show low or inconsistent loadings and explain little variance, yet are interpreted as meaningful clusters. These should be presented as exploratory patterns, not stable factors.
5. **Suitability of Cronbach’s alpha:**
  Cronbach’s alpha is presented as assessing “suitability for factor analysis,” but alpha measures internal consistency—not suitability. This needs correction in the Methods.

**Required for scientific soundness:**

 ✔ Correct the eigenvalue criterion to >1

 ✔ Add KMO and Bartlett’s test statistics

 ✔ Specify rotation method

 ✔ Reinterpret Factors 2–3 more cautiously as exploratory

 ✔ Clarify the proper use of Cronbach’s alpha

**3. Detail and Replicability of Methods**

**Rating: Partly**

**Strengths**

- Methods section outlines data collection timeframe and sampling context.
- Survey instrument was based on OECD frameworks.
- Data availability on Zenodo is excellent.

**Weaknesses**

1. **Survey instrument not adequately described:**
  - No sample survey items are provided.
  - Response scales (e.g., Likert scale values) are missing.
  - There is no explanation of how variables were selected for factor analysis.
2. **Analytical details are insufficient for replication.**
  Missing elements include:
  - Complete factor loadings
  - Variance explained by each factor
  - Communalities
  - Scree plot rationale
  - Details on how missing data (if any) were handled
3. **Sampling process not fully described:**
  - How were participants recruited?
  - What was the response rate?
  - Which ministries/agencies were included or excluded?

**Required for scientific soundness:**

 ✔ Add full description of survey items and scales

 ✔ Provide full factor loadings + variance explained

 ✔ Add KMO, Bartlett’s test, rotation method, communalities

 ✔ Describe participant recruitment and response rate

**4. Availability of Underlying Data and Materials**

**Rating: Yes**

**Strengths**

- All underlying data are openly accessible on Zenodo under a CC BY 4.0 licence.
- Supplemental materials (appendices, information sheets) are available and transparent.

**No revisions required.**

**5. Appropriateness and Interpretation of Statistical Analysis**

**Rating: Partly**

**Strengths**

- PCA-based factor analysis is reasonable for identifying patterns.
- Descriptive analysis aligns with the study's goals.

**Weaknesses**

1. **Technical errors in factor analysis (eigenvalue criterion, lack of diagnostics).**
2. **Overinterpretation of weak factors:**
  Factors 2–3 do not present robust latent structure.
3. **Financial implications sometimes overstated:**
  Although revised, some statements still imply fiscal impact without empirical measurement.

**Required for scientific soundness:**

 ✔ Apply correct factor-retention criteria

 ✔ Add factorability diagnostics

 ✔ Reframe weak factors as exploratory

 ✔ Ensure all fiscal claims are clearly stated as perceptions or international evidence—not measured outcomes

**6. Support of Conclusions by Results**

**Rating: Partly**

**Strengths**

- Conclusions about digitalisation coverage, skills development, and barriers are well supported.
- Limitations are thoroughly acknowledged.

**Weaknesses**

1. **Residual drift toward fiscal claims:**
  Some statements still imply cost savings or improved fiscal health without actual financial data.
2. **Causal claims should be avoided:**
  The study is perception-based and cross-sectional, and cannot draw causal conclusions.
3. **Policy recommendations require stronger linkage to findings:**
  For example, recommendations on digital skills or interoperability could more explicitly reflect the factor-analysis insights.

**Required for scientific soundness:**

 ✔ Ensure all fiscal and efficiency claims are clearly attributed to perception or literature

 ✔ Avoid causal language

 ✔ Strengthen empirical grounding for policy recommendations

**Overall Recommendation**

**Status: Major Revisions Required**

 The study presents valuable descriptive insights and the topic is highly relevant for policymakers and scholars. However, the paper requires substantial methodological and analytical corrections before it can be considered scientifically sound.

**Summary of Required Revisions (Must Be Addressed)**

**Methodology**

- Correct eigenvalue criterion to >1
- Add KMO and Bartlett’s test
- Specify rotation method
- Provide full factor loadings and variance explained
- Reinterpret weak factors as exploratory
- Clarify survey items, scales, and sampling procedures
- Correct misuse of Cronbach’s alpha

**Results & Interpretation**

- Reorder sections for clarity
- Ensure consistent framing of financial implications as perceptions
- Strengthen analytical discussion of digital ID findings
- More clearly link barriers → policy implications

**Literature & Structure**

- Reorganize review into conceptual themes
- Add stronger theoretical frameworks
- Strengthen link between literature and findings

If applicable, is the statistical analysis and its interpretation appropriate?

Partly

Is the study design appropriate and is the work technically sound?

Partly

Is the work clearly and accurately presented and does it engage with the current literature?

Partly

Are the conclusions drawn adequately supported by the results?

Partly

Are sufficient details of methods and analysis provided to allow replication by others?

Partly

Are all the source data and materials underlying the results available?

Yes

Reviewer Expertise:

My areas of research include digital government, public-sector digital transformation, information systems, digital public policy, AI-enabled governance, e-government evaluation frameworks, digital inclusion, and technology-driven public-sector reform. I also work on enterprise analytics, ICT policy assessment, and the socioeconomic impacts of digitalisation in emerging economies, particularly in Southeast Asia.

I confirm that I have read this submission and believe that I have an appropriate level of expertise to confirm that it is of an acceptable scientific standard, however I have significant reservations, as outlined above.

# Reviewer report: Reviewer response for version 1

**DOI:** 10.21956/openreseurope.21494.r58244

**Published:** 2025-09-12

**Authors:** Konstantinos Ioannou

**Affiliations:** 1 Department of Digital Systems, University of Peloponnese, Sparta, Lakonia, Greece

The study is a comprehensive and well-structured analysis of public sector digitalization in Thailand, with a clear research objective and a solid theoretical framework. It combines an extensive literature review with the analysis of primary data from a survey of 288 public sector representatives, which adds validity and practical depth to the findings. The use of factor and descriptive analysis is methodologically appropriate, while the presentation of results is clear and supported by statistical reliability indicators, such as Cronbach’s alpha. The study highlights significant progress in the adoption of digital tools and benefits of using them (efficiency, transparency, cost reduction), and  the challenges (digital divide, inequalities, insufficient evaluation of financial benefits). However, the sample is not fully representative, as local government —which constitutes a large and important part of government—is absent, and the reliance on officials’ perceptions may introduce subjectivity. Nevertheless, the study provides valuable insights for policymaking and can serve as a basis for comparative research in other countries. Overall, it represents an interesting contribution to the study of digital governance in Southeast Asia.

If applicable, is the statistical analysis and its interpretation appropriate?

Yes

Is the study design appropriate and is the work technically sound?

Yes

Is the work clearly and accurately presented and does it engage with the current literature?

Yes

Are the conclusions drawn adequately supported by the results?

Yes

Are sufficient details of methods and analysis provided to allow replication by others?

Yes

Are all the source data and materials underlying the results available?

Yes

Reviewer Expertise:

Digital Transformation, Telecommunications, Networks, Cybersecurity,

I confirm that I have read this submission and believe that I have an appropriate level of expertise to confirm that it is of an acceptable scientific standard.

# Reviewer report: Reviewer response for version 1

**DOI:** 10.21956/openreseurope.21494.r57460

**Published:** 2025-09-05

**Authors:** Oana Staiculescu

**Affiliations:** 1 Universitatea din Craiova, Craiova, Romania

**1. Is the work clearly and accurately presented and does it engage with the current literature?  ** ☒Yes 

 The article is well structured, clearly written, and provides a solid overview of the international literature (OECD, UN, UNDP, examples from Denmark, South Korea, Germany, etc.). The authors place the Thai case within the broader context of public sector digitalisation in Southeast Asia and highlight global challenges such as digital inclusion, data security, and open governance.

**Strengths:**

 • The literature review is extensive and includes comparative perspectives, which adds credibility.

 • Contextualisation in relation to national strategies (Thailand 4.0, Digital Plan 2024) is appropriate.

 • The text is readable and well-argued, with a strong balance between theoretical foundations and empirical data.

**2. Is the study design appropriate and is the work technically sound? ** ☒ Partly

 The study design is appropriate for the stated objectives: the authors used a survey applied to 288 respondents from government institutions and conducted a factor and descriptive analysis to identify the main areas of digitalisation. The methodology is described in detail, ensuring transparency and replicability.

**Strengths:**

 • The choice of survey method is justified by the aim of capturing perceptions and experiences.

 • The factor analysis is correctly applied and statistically validated (Cronbach’s coefficient).

 • Data are presented transparently, including their limitations.

**Limitations and recommendations:**

 • **Sample representativeness:** 85.76% of respondents (247 government agencies) come from central institutions, 14.24% (41 government agencies) from regional institutions, and the local level is completely absent. This limits the generalisation of results across the entire public system. The analysis could be positioned as valid for the central and regional levels only, with future research needed to capture local government aspects. It is also recommended to remove the null line from Table 1 (“Survey sample representation of government agencies”).

 • **Interpretation of factors:** The naming of factors is not explicitly provided in the article. From the authors’ explanations, one can infer the policy domains they refer to, but for greater clarity and interpretive value, each factor should be explicitly labelled and discussed. In addition, Factors 2 and 3 are briefly described and their contribution to understanding the phenomenon is unclear. The authors should develop this interpretation, explaining why they are less relevant.

 • **Link with financial impact:** Theoretical aspects regarding potential savings and spending efficiency are not directly measured, although they are mentioned in the “Results” section.

**4. Are all the source data and materials underlying the results available? ** ☒Yes 

 The authors provide raw data and related documentation through the Zenodo platform, in line with Open Research Europe transparency standards. The datasets (Excel and PDF files) and the survey instrument are accessible, allowing replication and verification of results. This is a strong point of the study.

**5. If applicable, is the statistical analysis and its interpretation appropriate?**  ☒ Partly

 The statistical analysis is based on factor and descriptive methods, which are appropriate for the type of data collected (survey). The authors report using principal component analysis and checking internal consistency with Cronbach’s alpha, which shows methodological rigour.

 However, a few points should be clarified:

 • **Secondary factors (2 and 3):** the loadings are lower and their interpretation is insufficiently detailed. In the current form, readers may perceive these results as marginal.

 • **Link to financial implications:** although the statistical analysis is correct, the data do not directly measure financial aspects. Assertions such as “digitalization reduces government costs” or “improves fiscal health” extrapolate from perceptions and general theories to economic-financial conclusions. For scientific rigor, since these are not directly quantifiable or demonstrated in the analysis, they should be presented as hypotheses or suggestions.

**6. Are the conclusions drawn adequately supported by the results? ** ☒ Partly

 The conclusions on the extension of digital tools across various domains (defense, environment, health, education) and on identified barriers (low digital literacy, urban–rural disparities, lack of data integration) are strongly anchored in the collected data and well presented.

**Positive aspects noted:**

 The “Discussion and conclusion” section provides a comprehensive overview of the digitalisation of Thailand’s public sector, integrated into the regional and global context. The conclusions are consistent with survey results, highlighting the policy areas most visibly affected by digitalisation and structural barriers such as digital literacy gaps and urban–rural infrastructure divides. A strong point is that the authors emphasise both the potential impact on social inclusion and the financial implications, as well as clear directions for future research.

**Aspects less well supported:**

 Some conclusions go beyond what the collected data can support. The financial impact, although relevant, is presented as a certainty even though the study does not directly measure savings or revenue increases. In addition, methodological limitations (a sample strongly skewed towards the central level) are only briefly mentioned, yet conclusions continue to be generalised to the entire public sector. Some ideas are also repetitive, and the policy recommendations remain fairly general, without detailed steps for implementation.

 Overall, the article represents a valuable and timely contribution to the field of public sector digitalisation, offering both an in-depth empirical perspective on Thailand and a solid integration into the broader Southeast Asian and global context. Despite some methodological limitations, the study demonstrates strong potential to inform both academic debates and policymaking. With minor clarifications and refinements—particularly in the interpretation of factors and the framing of financial implications—the article can become a reliable reference point for researchers and decision-makers seeking to understand and advance digital transformation in the public sector. Its emphasis on inclusion, transparency, and innovation highlights the transformative role that digitalisation can play in building more efficient and equitable societies.

If applicable, is the statistical analysis and its interpretation appropriate?

Partly

Is the study design appropriate and is the work technically sound?

Partly

Is the work clearly and accurately presented and does it engage with the current literature?

Yes

Are the conclusions drawn adequately supported by the results?

Partly

Are sufficient details of methods and analysis provided to allow replication by others?

Yes

Are all the source data and materials underlying the results available?

Yes

Reviewer Expertise:

Public Administration, Digitalization, E-democracy, Public Management, Leadership, Public Sector Accounting, and Economics.

I confirm that I have read this submission and believe that I have an appropriate level of expertise to confirm that it is of an acceptable scientific standard, however I have significant reservations, as outlined above.

# Response

**Published:** 2025-11-20

**Authors:** Estera Szakadatova

**Affiliations:** University of Economics in Bratislava, Bratislava, Bratislava Region, Slovakia

Thank you very much for your constructive and detailed comments on our manuscript. We greatly appreciate your feedback, we have revised the paper to address each of your suggestions.
